# Dynamic Lipidomic Responses to Inflammation and Physical Insult: A Comparative Review Across Blunt Force Trauma, Thermal Burn Injury, and Viral Infection

**DOI:** 10.1017/erm.2026.10038

**Published:** 2026-03-02

**Authors:** Harrison Szemray, Nathan Geoffrey Lawler, Samantha Lodge, Julien Wist, Luke Whiley

**Affiliations:** 1 Centre for Computational and Systems Medicine, Murdoch University, Murdoch, Perth, Australia; 2 School of Medical, Molecular and Forensic Science, Murdoch University, Murdoch, Perth, Australia; 3 School of Allied Health (Exercise Science), Murdoch University, Murdoch, Perth, Australia; 4 Chemistry Department, Universidad del Valle, Cali, Colombia; 5 Department of Metabolism, Digestion and Reproduction, Faculty of Medicine, Imperial College London, South Kensington, London, UK; 6 Curtin Medical Research Institute, Curtin University, Bentley, Perth, Australia; 7 School of Diagnostic and Therapeutic Sciences, Curtin University, Bentley, Perth, Australia; 8 Dementia Centre of Excellence Enable Institute, Curtin University, Bentley, Perth, Australia

**Keywords:** brain injuries, burns, COVID-19, human body, lipidomics, lipid metabolism, lipoproteins, magnetic resonance spectroscopy, mass spectrometry, traumatic

## Abstract

Acute insults ranging from blunt force trauma and thermal injury to pathogenic infection elicit systemic inflammatory cascades intended to limit further tissue damage. These responses are accompanied by metabolic disturbances that generate distinct biochemical signatures measurable through advanced analytical platforms, such as mass spectrometry and nuclear magnetic resonance spectroscopy (NMR). Although numerous studies have examined these metabolic alterations, findings remain fragmented across clinical and analytical disciplines, leaving it unclear whether the systemic metabolic response to acute insult is fundamentally conserved or insult-specific. In this comparative review, we consolidate evidence across diverse injury and infection contexts to identify shared metabolic patterns, context-dependent differences, and critical gaps in current understanding. Here, we focus on lipid and lipoprotein profiling of blood plasma and serum. We present exemplar case studies spanning traumatic brain injury, burn injury, and SARS-CoV-2 infection to illustrate how lipid and lipoprotein perturbations differ or converge across insult types. Notable observations include consistently elevated palmitic acid (16:0) and reduced phosphatidylcholine species across all three conditions, suggesting these features may represent cross-condition biomarkers and highlighting the value of comparative metabolic profiling. By integrating evidence across diverse contexts, we propose a framework describing the interplay between lipid metabolism, lipoprotein dynamics, and inflammatory activation. Finally, we discuss the translational potential of metabolic phenotyping in enhancing patient stratification, refining prognostic modelling, and improving patient outcomes.

## Introduction

This review addresses whether systemic metabolic responses to different acute insults are convergent, reflecting a shared inflammatory response, or divergent, exhibiting insult-specific patterns, and considers how these patterns inform clinical management. Inflammatory processes are part of the body’s defence mechanism against injury or pathogenic infection (Ref. 1). These processes entail the activation of diverse immune cells, the release of proinflammatory cytokines and the generation of reactive oxygen species (ROS) and are typically characterised by localised swelling, pain and redness due to increased vascular permeability (Ref. 1). Inflammatory conditions are broadly classified into acute and chronic inflammation; the progression of acute to chronic inflammation is known as subacute inflammation. Acute inflammation ensues immediately after a trauma, injury or infection, causing the release of soluble mediators, such as cytokines and chemokines, to promote the migration of neutrophils and macrophages to the site of injury. These cells are part of the natural innate humoral response that can be active in acute inflammation (Ref. 2). If the acute inflammatory response does not resolve the damage at the injury site, a transition to subacute inflammation occurs that can last from 2 to 6 weeks. Furthermore, chronic inflammation can persist for months to years following injury, exposing the host to collateral damage of healthy tissues and cells through a persistent and non-resolving low-grade inflammation activated by damage-associated molecular patterns (Ref. 2). Clinically, failure to resolve inflammation complicates patient stratification and the timing of interventions. Additionally, a persistent state of chronic inflammation is linked with the development of chronic inflammatory diseases, such as cardiovascular disease, diabetes, Alzheimer’s disease, chronic lung disease and cancer (Ref. 3), emphasising the need for more mechanism-informed biomarkers to guide patient treatment.

When studying the acute inflammatory response of the human system, research has traditionally focused on protein biomarkers of immunological and inflammatory functions, such as C-reactive protein (CRP), erythrocyte sedimentation rate, procalcitonin and white blood cell counts that are established clinical measurements (Ref. 4). However, such protein responses are also accompanied by changes in lipid metabolism (Ref. 5). Lipids are a diverse class of biomolecules that play essential roles in numerous biological processes, such as energy storage and membrane formation (Refs 6, 7), as well as regulating membrane fluidity and permeability, and participating in cellular signalling pathways (Refs 8, 9). Lipids are readily accessible in whole blood, serum or plasma, and therefore hold great promise as complementary markers for monitoring systemic inflammation with greater granularity (Ref. 10). However, most studies focus on a single type of inflammatory insult compared to a control group to identify lipidomic biomarkers. This approach often overlooks the plausibility of a typical systemic lipidomic response that may occur in response to different inflammatory insults. Notably, transcriptomics and genomic analyses by Xiao and colleagues reported nearly identical physiological responses at both the transcriptomic and genomic levels following blunt force and thermal insults, despite an ‘apparent’ disparity in the cause of injury (Ref. 11). These responses also mirror those to severe viral infections, such as sepsis and systemic inflammatory response syndrome (Refs 11–13). Together, these observations motivate a comparative approach to test whether lipidomic responses converge across distinct injuries or diverge in context-dependent ways.

Here, we conduct a narrative review of the literature to document lipidomic and lipoprotein responses across multiple insults that elicit systemic inflammation in the human body. We focus on metabolic phenotyping of blood plasma and serum, with emphasis on lipid and lipoprotein profiling using mass spectrometry and nuclear magnetic resonance. We aim to identify patterns common across insults versus those specific to insults, and to link these patterns to clinical challenges associated with systemic inflammation. To achieve this, we selected exemplars frequently cited in the literature that represent a diverse spectrum of insults, capturing acute physical trauma, thermal burn injury and viral infection. The review is organised as follows: we first outlined above the biological and analytical foundations of lipid and lipoprotein phenotyping in systemic inflammation; we then present the three exemplar case studies to compare convergent and divergent lipidomic signatures; next, we integrate these findings into a conceptual framework linking lipid metabolism, lipoprotein dynamics and the inflammatory cascade, before finally discussing implications for patient stratification, prognostic modelling and targeted interventions to mitigate inflammation and improve clinical outcomes.

## Selection of exemplar inflammatory insults

**Figure 1.**
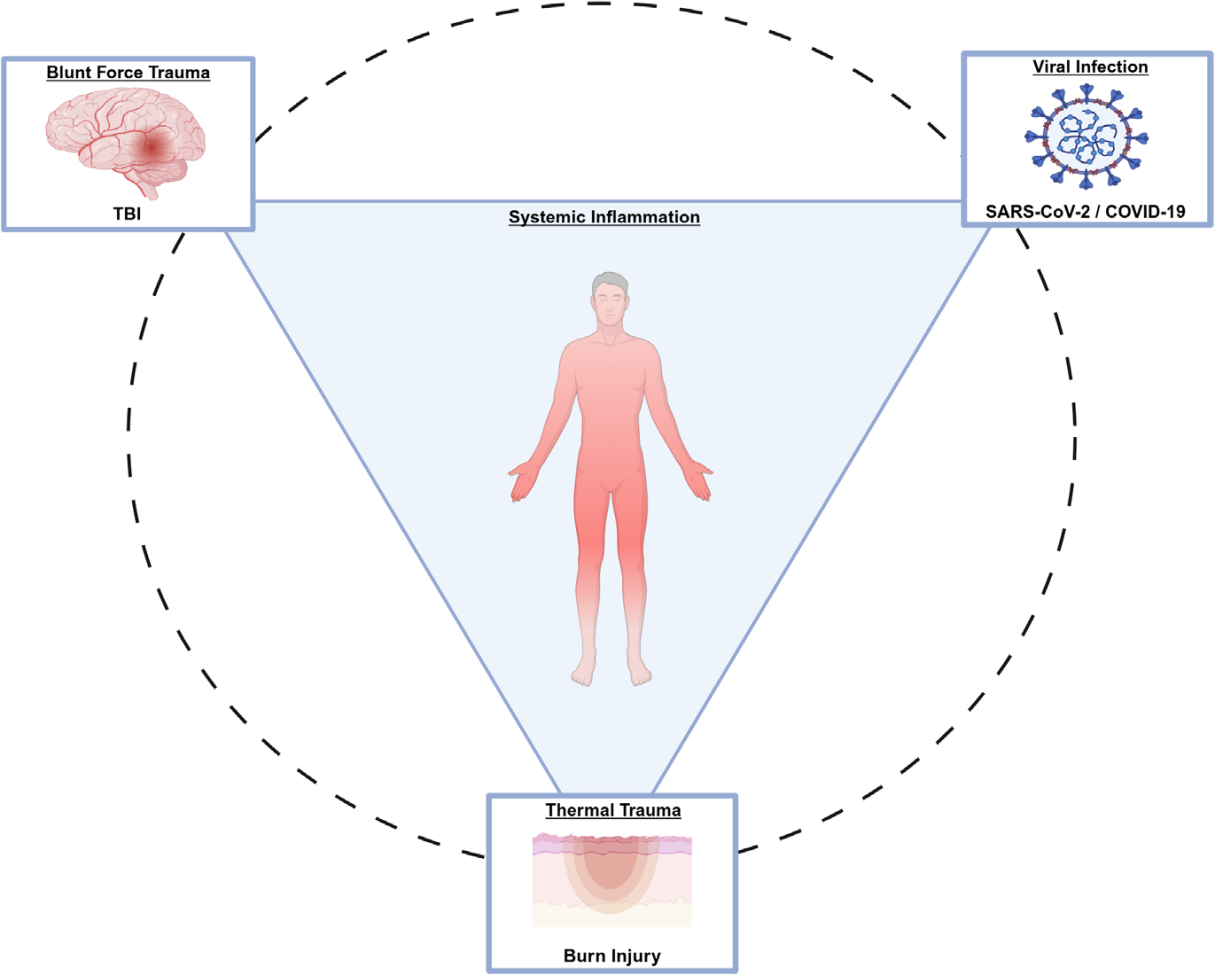
Literature review scope. Illustrates the scope of the literature review, with systemic inflammation as the primary theme and how each model of acute insult causes a systemic inflammatory response. Created in BioRender. Szemray, H. (2026) https://BioRender.com/lc6zhk4.

We elected to focus the review on the systemic response to specific acute injury exemplars within each acute insult category, aiming to narrow the focus and elucidate the standard and divergent metabolic signatures reported in the literature. For blunt force injury, we utilised literature on traumatic brain injury (TBI), as it is the most observed localised blunt force trauma (Ref. 16). For thermal injury-related insults, we utilised literature on heat-induced burn injuries, the most common source of burn injury (Ref. 14). For comparisons with viral insults, we used literature exemplars of viral infections, such as those caused by the severe acute respiratory syndrome coronavirus 2 (SARS-CoV-2), resulting in coronavirus disease 2019 (COVID-19), due to the plethora of recent literature. These exemplars were also chosen because each affects a distinct region of the body: TBI impacts the brain, burn injuries affect the skin and SARS-CoV-2 targets the respiratory system. This selection was intended to provide a holistic view of the systemic inflammatory response across diverse physiological domains (visualised in Figure 1).

For this review, we queried the literature to capture the systemic lipidomic response to acute inflammatory insults using three predefined exemplars: TBI, heat-induced burn injury and SARS-CoV-2/COVID-19. Databases and search engines such as Google Scholar, PubMed and Scopus were utilised to find literature using a standard search string. The search string applied was as follows: ‘(“TBI” OR “traumatic brain injury” OR “burns” OR “burn injury” OR “SARS-CoV-2” OR “COVID-19”) AND (“lipidomics” OR “lipid metabolism” OR “metabolic phenotyping”) AND (“mass spectrometry” OR “magnetic resonance spectroscopy”) AND (“plasma” OR “blood plasma” OR “serum” OR “blood serum”)’. Inclusion criteria consisted of original studies reporting systemic lipidomic or lipid metabolic profiling of plasma/serum in the context of the specified acute insults, using mass spectrometry or magnetic resonance spectroscopy (discovery or targeted), with sufficient methodological detail to interpret lipid species or pathway-level changes. We excluded studies focused exclusively on non-systemic matrices (e.g., tissue, cerebrospinal fluid and blister fluid) without accompanying plasma/serum data, injuries outside the three exemplar categories, studies with no control cohort and studies reporting total lipid class concentrations.

The availability of lipidomic literature varies across the aforementioned examples. SARS-CoV-2/COVID-19 currently has the most extensive plasma/serum lipidomic evidence, driven by the rapid growth of studies since 2020, enabling more granular comparisons by severity and time course. TBI presents a moderate evidence base, with at least nine studies reporting systemic lipidomic perturbations, though cohorts and analytical platforms are heterogeneous. Burn injury has comparatively fewer plasma/serum lipidomic studies; historically, investigations have focused on tissue and blister fluid, with systemic lipidomics emerging only more recently. Readers should therefore expect some asymmetry in data completeness across sections, with COVID-19 providing denser evidence, TBI moderate coverage and burns a more limited but growing systemic lipidomic literature.

### Blunt force trauma exemplar: TBI

Blunt force trauma, also referred to as non-penetrating or blunt trauma, is a type of injury that happens when an external force, such as acceleration, deceleration, shearing or crushing, impacts the human body (Ref. 15). This type of injury is commonly seen in medical examinations following incidents such as road accidents, physical assaults, sports-related injuries and falls, particularly among the elderly. Depending on the area of the body affected, blunt force trauma can be further categorised into blunt abdominal trauma, blunt thoracic trauma and blunt cranial trauma (Ref. 15). Physical outcomes can range from single-organ to multi-organ trauma. The most observed blunt force trauma and hence selected as an exemplar focus of this review, are blunt cranial traumas, commonly referred to as TBI.

TBI can cause significant cognitive, psychological and physical health complications and impose a substantial socioeconomic burden (Ref. 15). In TBIs, shear, direct and rotational forces disrupt normal cellular functions and are categorised into acute and secondary injuries. Acute TBI injuries can cause epidural haematoma, subdural haematoma, subarachnoid haemorrhage and intraventricular haemorrhage, resulting in vasculature, neural and glial tissue injury (Ref. 16). Tissue injury triggers an immediate and prolonged inflammatory response, activating microglia and astrocytes. These cells release proinflammatory cytokines, chemokines and ROS, causing neuronal damage and cell death (Ref. 17). The acute injury is followed by a secondary phase (chronic TBI), the leading cause of TBI-related hospital deaths, causing cellular and molecular brain damage and occurring days to months after the initial injury (Ref. 18). The secondary injury is characterised by an influx of immune cells, including neutrophils and monocytes, to the site of injury (Ref. 17). The most prevalent events during the secondary injury are blood–brain barrier disruption and cell death (Ref. 19). The inflammatory response following a TBI is a complex process involving numerous cells and signalling pathways. Among these are lipid-derived molecules, such as eicosanoids, docosanoids and prostaglandins, which are metabolites of polyunsaturated fatty acids (PUFAs) and regulate inflammation, pain and fever after injury (Refs 20–23). Numerous studies investigating TBI have reported on alterations in metabolic profiles, with nine specifically documenting lipidomic perturbations. These findings collectively support the hypothesis that TBI elicits a complex and dynamic lipidomic response (Refs 24–32).

### Thermal trauma exemplar: Burn injury

Burn injury damage is commonly caused by thermal, electrical or chemical agents, which initiate a chain reaction that results in tissue inflammation and further damage. The complications associated with burn injuries include infection, organ dysfunction and mortality (Ref. 33). The systemic inflammatory response is crucial to the pathophysiology of burn injury, inducing a proinflammatory environment characterised by significantly elevated levels of cytokines, immune cells and lipid mediators of inflammation (Refs 34, 35). Inflammation resulting from burn injury can be divided into two phases: an acute phase that occurs immediately in the days post-injury and a chronic phase that can persist from months to years post initial injury (Ref. 36). In response to the injury, the acute phase is characterised by the release of proinflammatory cytokines and chemokines (Ref. 37). The chronic phase activates immune cells, generates ROS and proinflammatory mediators and recruits additional immune cells to the injury site (Ref. 38). A complex interaction between local tissue injury and the systemic inflammatory response characterises the pathophysiology of burns (Ref. 39). Immediately following a burn injury, an inflammatory response is triggered both locally and systemically at the injury site, a process that is not yet fully understood. While most of the available literature focuses on the use of tissue and burn blister fluid, recent research has turned to blood serum and plasma lipidomics to analyse the systemic lipid response to burn injury, with many systemic changes observed (discussed in more detail below) (Refs 14, 36, 40).

### Viral infection exemplar: SARS-CoV-2 (COVID-19)

Viral infection can cause a considerable physical and physiological burden on the human system. The recent high-profile global epidemic of the novel coronavirus, SARS-CoV-2, provides such an exemplar, with infection resulting in COVID-19. The disease predominantly affects the respiratory system and ranges from moderate to severe respiratory distress; however, severe examples result in systemic implications, including multiple organ failure. In addition to infecting the respiratory system, the virus has also been shown to infect the gastrointestinal tract, particularly in children (Refs 41, 42). Pathogenesis of COVID-19-induced inflammation depends on immune response, which is influenced by factors such as age, body mass indexand overall health (Refs 43, 44). The pathophysiology of COVID-19 is characterised by a systemic immune response that is dysregulated and characterised by the release of proinflammatory cytokines, chemokines and ROS. In severe instances, a cytokine storm can contribute to the development of acute respiratory distress syndrome, sepsis and multi-organ failure (Ref. 45). A cytokine storm is defined as an unregulated release of high concentrations of cytokines by the innate immune system, causing a systemic hyperinflammatory state (Ref. 46).

Viral infection of respiratory epithelial cells results in the release of inflammatory cytokines and chemokines, such as interleukin IL-1β, IL-6, tumour necrosis factor (TNF) and C–C motif chemokine ligand 2 (CCL2) (Ref. 47). Infection with SARS-CoV-2 triggers a two-stage response from the immune system. The first stage is the innate immune response, where innate immune cells secrete cytokines and interferons that activate the adaptive immune response. During this stage, COVID-19 symptoms, such as sore throat, cough, myalgia and headache, are the most reported (Ref. 48). However, not all individuals infected with SARS-CoV-2 will exhibit symptoms. Of note for this review, patients with preexisting comorbidities, such as dyslipidemia, have been reported to be at much greater risk of severe COVID-19 symptoms (Ref. 49). Changes in the phospholipids, sphingolipids and eicosanoids have been observed in the lipid profile of COVID-19 patients (Refs 50–53). Furthermore, Lodge and colleagues demonstrated that as COVID-19 severity increases, so does the number of perturbed lipid and lipoprotein species (Ref. 54). The highly inflammatory systemic response to SARS-CoV-2 infection (COVID-19) makes SARS-CoV-2 a viable model for exploring the complex relationship between different types of acute insults.

## Comparison of the systemic lipidomic response to TBI, burn injury and SARS-CoV-2 infection

**Figure 2.**
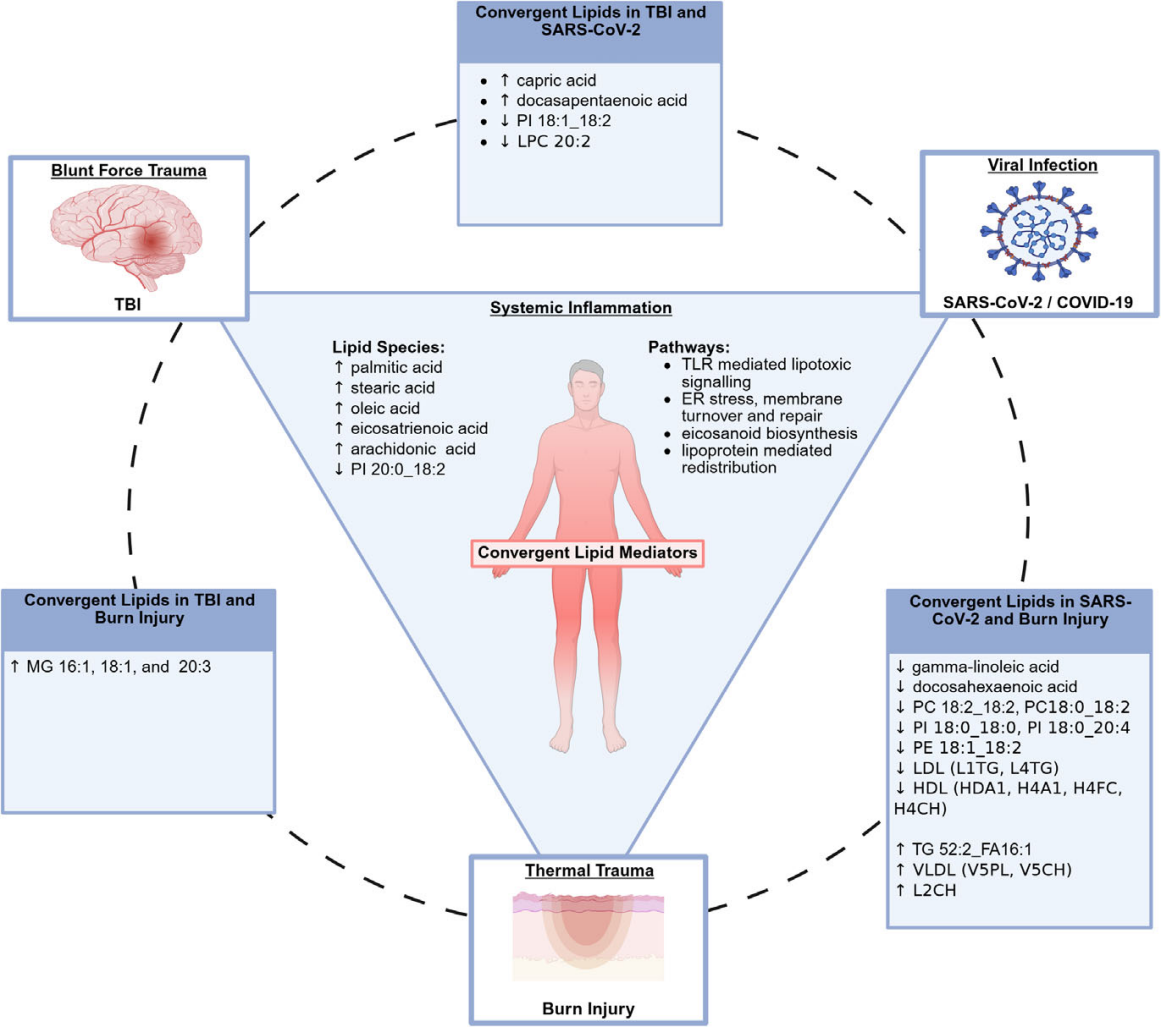
Summary of the key findings comparing lipidomic responses to TBI, burn injury and SARS-CoV-2 infection, synthesised from Tables 1–5. ↑: increased levels; ↓: decreased levels; MG: Monoacylglycerol; TG: Triacylglycerol; PC: Phosphatidylcholine; PI: Phosphatidylinositol; LPC: Lysophosphatidylcholine; PE: Phosphatidylethanolamine; LDL: Low-Density Lipoprotein; L1TG: Low-Density Lipoprotein-1 Subclass Triglycerides; L4TG: Low-Density Lipoprotein-4 Subclass Triglycerides; L2CH: Low-Density Lipoprotein-2 Subclass Cholesterols; HDL: High-Density Lipoprotein; HDA1: HDL Apolipoprotein A-I; H4A1: High-Density Lipoprotein-4 Subclass Apolipoprotein-A1; H4FC: High-Density Lipoprotein-4 Subclass Free Cholesterol; H4CH: High-Density Lipoprotein-4 Subclass Cholesterols; VLDL: Very Low-Density Lipoprotein; V5PL: Very Low-Density Lipoprotein-5 Subclass Phospholipids; V5CH: Very Low-Density Lipoprotein-5 Subclass Cholesterols. Created in BioRender. Szemray, H. (2026) https://BioRender.com/ekp56hy.

This review aims to identify both commonalities and discrepancies in systemic lipidomic profiles observed across blunt force trauma, burn injury and viral infection, with a focus on perturbations in lipid metabolism. Specifically, we examine changes in lipid species within blood serum or plasma associated with TBI, burn injury and SARS-CoV-2 infection when compared to healthy controls, each representing a distinct category of acute insult. To streamline the review, we present tables highlighting lipid species that are altered in two or more of these conditions, providing a consolidated view of lipidomic changes associated with inflammation across different types of trauma. A summary of key findings has been included below in Figure 2.

A major challenge in compiling this review is the lack of standardisation in how lipid species are reported in the literature (Ref. 55). This issue becomes particularly evident when considering the lipid side chain composition. For instance, a lipid may be reported in a simplified form, such as PC 36:4, yet a database search (e.g., LipidMaps) for PC 36:4 can return up to 27 structurally distinct molecular species (e.g., PC(14:0/22:4(7Z,10Z,13Z,16Z))) (Ref. 56). Such oversimplification complicates cross-study comparisons and may obscure biologically significant findings. In this review, we treat summed sidechain lipid species as individual metabolites without making assumptions about their precise structural identities.

### Fatty acids (free fatty acid)

FAs are fundamental components of more complex lipid species. FAs are composed of a hydrocarbon chain with a carboxyl group that is classified as either unsaturated (with one or more double bonds) or saturated (no double bonds) (Ref. 57). Under normal metabolic conditions, FAs’ primary roles are as an energy source via oxidation when the glucose supply is low and as components of cellular membranes. However, FAs also have structural and signalling roles, including as precursor molecules for lipid mediators (Ref. 58), with the release and uptake of FAs from cellular membranes having specific signalling, metabolic or functional roles (Ref. 59). In an inflammatory state, FAs activate inflammatory mechanisms through intracellular and cell surface receptors/sensors that control inflammatory signalling and gene expression (Ref. 60). Alterations in FA concentrations have been consistently observed in the literature in the study of TBI, burn injury and SARS-CoV-2 infection (Table 1).

**Table 1.** Significantly altered fatty acid concentrations compared to controls across blunt force trauma, thermal injury, and viral infection models

Subclass	Species	Force trauma (TBI)	Thermal trauma (burn injury)	Viral infection (SARS-CoV-2)
Saturated fatty acid	Capric acid (FFA 10:0)	↑ (Ref. 61)	NR	↑ (Ref. 62)
	Palmitic acid (FFA 16:0)	↑ (Ref. 27)	↑ (Refs 40, 63)	↑ (Refs 62, 64)
	Stearic acid (FFA 18:0)	↑ (Refs 26, 27)	↑ (Refs 35, 63)	↑ (Ref. 64)
Monounsaturated fatty acid	Oleic acid (FFA 18:1)	↑ (Ref. 27)	↑ (Refs 35, 40, 65)	↑ (Refs 50, 62) ↓ (Refs 52, 64)
Polyunsaturated fatty acid	Linoleic acid (FFA 18:2)	↑ (Ref. 31)	↑ (Ref. 35)	↑ (Refs 50, 62, 64) ↓ (Ref. 65)
	Gamma-linolenic acid (FFA 18:3)	↑ (Ref. 66)	↓ (Ref. 65)	↓ (Refs 62, 64)
	Eicosatrienoic acid (FFA 20:3)	↑ (Ref. 66)	↑ (Ref. 65)	↑ (Refs 62, 64)
	Arachidonic acid (FFA 20:4)	↑ (Refs 27, 31)	↑ (Ref. 65) ↓ (Ref. 14)	↑ (Refs 50, 62, 64)
	Docosapentaenoic acid (FFA 22:5)	↑ (Ref. 27)	↓ (Ref. 14)	↑ (Ref. 67)
	Docosahexaenoic acid (FFA 22:6)	↑ (Ref. 27)	↓ (Ref. 14)	↓ (Ref. 54)

*Note:* ↑: increased levels; ↓: decreased levels; NR: not reported in cited literature; FFA: free fatty acid. To streamline the focus of this review, lipidome alterations observed in two or more insult types are tabularised above.

#### Linoleic acid pathway

**Figure 3.**
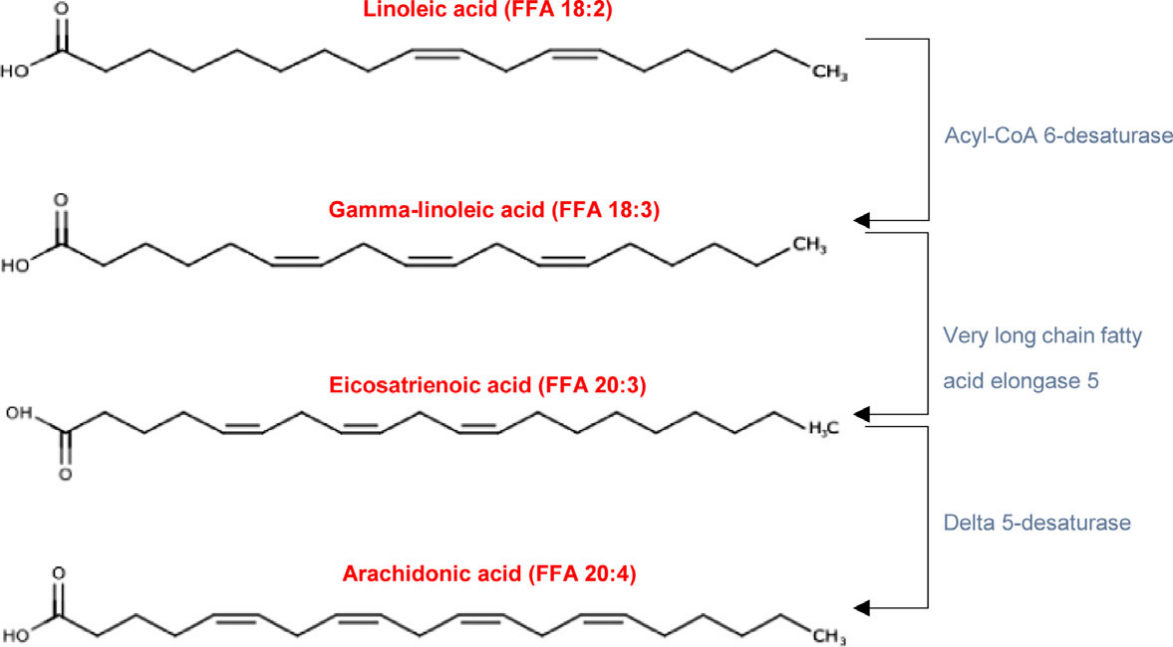
Conversion of Linoleic acid to longer-chain polyunsaturated fatty acids under inflammatory conditions. The names of free fatty acid species are displayed in red, while enzyme names are displayed in blue.

The literature demonstrates an agreement in reported associations of PUFAs: linoleic acid (free FA (FFA) 18:2), eicosatrienoic acid (FFA 20:3) and arachidonic acid (FFA 20:4), with discrepancies in the reporting of gamma-linolenic acid (FFA 18:3) (elevated in TBI, but decreased in burn and viral infection). PUFAs are known eicosanoid precursors and have been shown to reside in the same metabolic pathway active during inflammation (Figure 3) (Ref. 68).

In the literature, plasma linoleic acid (FFA 18:2) has been demonstrated to increase in concentration for TBI (Ref. 31), burn injury (Ref. 35) and SARS-CoV-2 infection (Refs 50, 62, 64), indicating a similar response across all three types of insult. However, it should be noted that linoleic acid concentration has also been reported to decrease in children who have experienced burn injury, indicating that the inflammatory lipid response may vary with age and differ between paediatric and adult populations (Ref. 65). The possible age-dependent differences in lipodomic profiles highlight the importance of standardising the reporting of metadata such as age and biological sex alongside biomarker reports to enable cross-study comparability. Linoleic acid has been associated with inflammation via the lipoxygenase pathway, as it forms derivatives known as hydroxyoctadecadienoic acids, oxo-hydroxyoctadecadienoic acids and epoxy-hydroxyoctadecadienoic acids, which are reported to hold key roles in the inflammatory response (Ref. 69, 70).

Gamma-linolenic acid (FFA 18:3) is reported to increase in TBI (Ref. 66), yet it has been shown to decrease in burn injury (Ref. 65), and SARS-CoV-2 (Ref. 62, 64). This discrepancy in systemic gamma-linolenic acid concentrations in response to the TBI is unclear, but it may indicate a difference in lipid response dependent on the initiating insult or be a reflection of neuroinflammation. Gamma-linolenic acid’s involvement in the inflammatory process extends to the tissue repair and wound-healing process, where it promotes tissue regeneration, collagen synthesis and angiogenesis, aiding in the resolution of inflammation and the healing of damaged tissues and cells (Ref. 71). Furthermore, gamma-linolenic acid can be metabolised by the elongation of very-long-chain FAs protein 5 to produce eicosatrienoic acid (FFA 20:3), which is discussed below.

Eicosatrienoic acid (FFA 20:3), also commonly referred to as dihomo-gamma-linolenic acid, has been reported to have significantly increased concentrations in TBI (Ref. 66), burn injury (Ref. 65) and SARS-CoV-2 infection (Ref. 62, 64). Under inflammatory conditions, eicosatrienoic acid is a precursor of arachidonic acid (FFA 20:4) via the action of the enzyme delta 5 FA desaturase (Ref. 68). Eicosatrienoic acid is also further converted to prostaglandin E1 (Ref. 68). Prostaglandin E1 exhibits anti-inflammatory actions by inhibiting the production of proinflammatory cytokines, including IL-1, TNF-α and IL-6 (Ref. 72).

Arachidonic acid (FFA 20:4) concentrations increased in TBI (Refs 27, 31) and SARS-CoV-2 viral infection (Refs 50, 62, 64) and burn injury (Ref. 65). However, Ryan and colleagues reported decreased concentrations in burn injury patients (Ref. 14). The decreased concentrations of thermal trauma observed by Ryan and colleagues may be due to the non-severe nature of burn injury in the study’s sample population (Ref. 14). Additionally, burn injury can induce systemic lipid peroxidation, resulting in the degradation and breakdown of arachidonic acid, which may contribute to the decreased concentration of arachidonic acid in blood (Ref. 73). Arachidonic acid plays a crucial role in the inflammatory response due to its conversion into various bioactive lipid mediators known as eicosanoids. These potent lipid mediators form a bioactive lipid network that has proven to be a highly complex pathway, exhibiting both proinflammatory and anti-inflammatory properties. We refer the reader to Calder’s work for a more thorough review of the association between deregulated eicosanoid pathways and various inflammatory diseases, such as cystic fibrosis, multiple sclerosis and acute respiratory syndrome (Ref. 74). Eicosanoids, such as prostaglandins, thromboxanes and leukotrienes, act as local hormones and induce diverse effects on the inflammatory process following trauma.

Prostaglandins are synthesised from arachidonic acid via the cyclooxygenase pathway and have pleiotropic effects on inflammation (Ref. 75). For example, prostaglandin E2 has been shown to promote inflammation by increasing vasodilation, vascular permeability and the recruitment of immune cells to the site of injury or infection (Ref. 75). Alternatively, prostaglandin D2 and prostacyclin have been demonstrated to exert anti-inflammatory properties by limiting immune cell activation and promoting vasodilation (Ref. 22). Thromboxanes are also produced from arachidonic acid via the cyclooxygenase pathway. For example, thromboxane A2 is a potent vasoconstrictor and platelet aggregator, promoting clot formation and vascular constriction at the site of injury (Ref. 76). While thromboxane A2 is essential for haemostasis and wound healing, upregulated production of thromboxane A2 can further promote inflammation and tissue damage. Leukotrienes are synthesised from arachidonic acid through the lipoxygenase pathway. They are potent proinflammatory mediators that contribute to increased vascular permeability, chemotaxis of immune cells and the release of inflammatory cytokines (Ref. 77). Under normal inflammatory conditions, the release and synthesis of eicosanoids are tightly regulated to ensure a balanced and appropriate immune response. However, dysregulation of arachidonic acid metabolism and eicosanoid production can lead to excessive inflammation and contribute to the progression of chronic disease (Ref. 78).

#### Palmitic acid

Palmitic acid (FFA 16:0) concentrations are reported to increase in patients with TBI (Ref. 27), burn injury (Refs 40, 63) and SARS-CoV-2 viral infection (Refs 62, 64). Qi et al. demonstrated that increased concentrations of palmitic acid in burn injury patients correlate with increased mortality, regardless of age, outcome or burn severity (Ref. 40). Elevated plasma or serum concentrations of palmitic acid have been linked to increased proinflammatory cytokine production via the activation of Toll-like receptor 2 (TLR2) and TLR4 signalling pathways (Ref. 79). Furthermore, products of palmitic acid metabolism increase ROS production, impact the activation of protein kinase C and affect endoplasmic reticulum (ER) stress (Ref. 79). The role of ROS species in inflammation and progression to chronic inflammatory diseases is well-characterised in the literature and has been extensively reviewed elsewhere (Ref. 80). Given these findings, palmitic acid may be considered a candidate biomarker for future lipidomic research.

#### Stearic acid

Stearic acid (FFA 18:0) concentrations increase in patients with TBI (Refs 26, 27), burn injury (Refs 35, 63) and SARS-CoV-2 viral infection (Ref. 64). Previous literature has demonstrated that incubation of human monocytic cells with stearic acid results in the upregulation of MIP-1α/CCL3 upon exposure to TNF-α via a myeloid differentiation factor 88 independent TLR4/interferon regulatory factor 3 signalling pathway. Furthermore, the synergy of stearic acid and TNF-α involves the nuclear factor-kappa B (NF-κB)/activator protein-1-mediated signalling pathway, which has been shown to regulate many proinflammatory genes (Refs 81, 82). In 2014, Mu and associates conducted a randomised controlled trial demonstrating significant associations between palmitic acid and stearic acid and CRP and IL-6, respectively (Ref. 83). These observations highlight palmitic acid and stearic acid as potential biomarker candidates, as CRP and IL-6 are two standardised markers of systemic inflammation. Moreover, Picod and colleagues demonstrated the efficacy of these biomarkers for evaluating the prognosis of critically ill patients (Ref. 84).

#### Oleic acid

Oleic acid (FFA 18:1) concentrations have been reported to increase in TBI (Ref. 27), burn injury (Refs 35, 40, 65) and SARS-CoV-2 infection (Refs 50, 62). However, it should be noted that oleic acid has also been reported to decrease in patients with SARS-CoV-2 infection (Refs 52, 64). The reasons behind this conflict in mean concentration shifts in the literature are unclear. The studies had contrasting collection time points in the SARS-CoV-2 infection timeframe, which may impact lipid concentrations due to the severity of the infection at the given sample time point. Oleic acid has been shown to attenuate inflammation by inhibiting the proinflammatory effects of stearic acid. Specifically, supplementation with oleic acid in human aortic endothelial cells reduces the incorporation of stearic acid in cells and inhibits the activation of NF-κB (Ref. 85). In 2019, Nakajima et al. also demonstrated that increased extracellular oleic acid strongly induced astrocytic lipid droplet accumulation, aiding survival against lipotoxic conditions in the central nervous system (Ref. 86). Furthermore, Bozza and Viola suggested that lipid droplets are inducible organelles involved in regulating lipid metabolism, cell signalling and the synthesis and secretion of inflammatory mediators, such as eicosanoids (Ref. 87), possibly highlighting oleic acid as a potent biomarker of the human system’s inflammatory status.

#### Capric acid

Capric acid (FFA 10:0) concentrations increased in both TBI (Ref. 61) and SARS-CoV-2 infection (Ref. 62). However, no literature was found reporting significant concentration changes in burn injuries. A study by Huang et al. demonstrated that capric acid at 100 mM inhibits inflammatory cytokines, such as TNF-α, IL-6 and IL-8, by inhibiting mitogen-activated protein kinase (MAPK) phosphorylation and NF-κB activation (Ref. 82). In 2012, Malapaka et al. demonstrated that capric acid occupies a novel binding site and binds to peroxisome proliferator-activated receptor-α (PPARα) and PPARβ/δ (Ref. 88). These findings suggest that capric acid modulates inflammatory gene expression by inhibiting the activation of MAPK phosphorylation, NF-κB and PPAR.

### Neutral glycerolipids (tri- di- and mono-acylglycerides)

Glycerolipids are formed by the esterification of FAs to glycerol and can be broken down further into monoacylglycerol (MG), diacylglycerol (DG) and triacylglycerol (TG) (Ref. 89). MG is well-characterised throughout the literature and is thought to be pivotal in mood, appetite, motor function, pain, learning and memory (Ref. 90). DGs function as cellular membrane components, raw building materials for glycerophospholipids and secondary lipid messengers (Ref. 91). TG is converted from excess glucose in the body to be stored for adenosine triphosphate production (Ref. 92), primarily utilised for energy storage and membrane structure (Ref. 93). Furthermore, high TG concentrations in the blood indicate increased blood glucose levels, a putative sign of insulin resistance and metabolic dysregulation (Ref. 94). Emerging evidence suggests a role for glycerolipids in modulating the inflammatory cascade. A significant issue with reporting glycerolipids in the literature is the strong preference for reporting concentration as a summed class rather than individual lipid species. Significantly altered blood lipid species concentrations for blunt force (TBI), thermal (burn injury) and viral infection (SARS-CoV-2) are summarised below (Table 2).

**Table 2.** Significantly altered glycerolipid concentrations compared to controls across blunt force trauma, thermal injury, and viral infection models

Subclass	Species	Force trauma (TBI)	Thermal trauma (burn injury)	Viral infection (SARS-CoV-2)
Monoacylglycerol (MG)	MG 16:1	↑ (Ref. 66)	↑ (Ref. 14)	↓ (Ref. 54)
	MG 18:1	↑ (Ref. 66)	↑ (Ref. 14)	↓ (Ref. 54)
	MG 20:3	↑ (Ref. 66)	↑ (Ref. 14)	↓ (Ref. 54)
	MG 20:4	NR	↑ (Ref. 14)	↓ (Ref. 54)
Diacylglycerol (DG)	DG 18:1_22:6	↑ (Ref. 27)	NR	↓ (Ref. 54)
Triacylglycerol (TG)	TG 52:2_FA 16:1	NR	↑ (Ref. 14)	↑ (Ref. 54)

Note: ↑: increased levels; ↓: decreased levels; NR: not reported in cited literature; MG: Monoacylglycerol; DG: Diacylglycerol; TG: Triacylglycerol. To streamline the focus of this review, lipidome alterations observed in two or more insult types are tabularised above.

#### Monoacylglycerol

The literature reports increases in MG species, including MG 16:1, MG 18:1 and MG 20:3, for TBI (Ref. 66) and burn injury (Ref. 14). In contrast, SARS-CoV-2 viral infection is associated with a decrease in MG species (Ref. 54). Literature reporting the specific role of these lipid species within blunt force trauma, thermal trauma and viral infection is currently limited, and even less information is known about their direct effects on the inflammatory process under these conditions. However, MGs play a crucial role in the endocannabinoid system, which is a potent regulator of the inflammatory process and is perturbed in conditions, such as chronic inflammation, metabolic disorders, cancer, autoimmune diseases and neuroinflammatory disorders. The reader is referred to recent reviews on this topic (Refs 6, 95).

#### Diacylglycerol

DG (DG 18:1_22:6) containing docosahexaenoic acid (FA 22:6) was reported to increase in concentration post TBI (Ref. 27), yet decreased in SARS-CoV-2 viral infection (Ref. 54). This differential regulation of DG 18:1_22:6 suggests that its metabolic pathways are modulated in distinct ways depending on whether the physiological stress arises from viral infection or force trauma. Understanding these variations could provide insights into the specific lipidomic responses for each condition. Investigating the correlation between DG species and FFAs containing the same species would provide valuable insight into whether the up- or downregulation of these species adversely affects FFA availability, highlighting possible implications for the inflammatory response following an acute insult.

#### Triacylglycerol

Reporting of significantly perturbed TG species in the TBI field was limited and thus is excluded from this review. Burn injury and SARS-CoV-2 viral infection have been reported to perturb numerous TG species significantly. Specifically, TG 52:2_FA 16:1 was found to increase in both burn injury (Ref. 14) and SARS-CoV-2 infection (Ref. 54). Ryan et al. reported that five TG species were significantly increased in burn patients (Ref. 14), while Lodge et al. demonstrated numerous TG species as significantly altered in SARS-CoV-2; however, when considering the associated FA side chains, the TG species displayed increased and decreased concentrations with no particular trend apparent (Ref. 54). Please note that these specific lipid species were not included in Table 2 due to not meeting the criteria of being in two or more insult types.

### Glycerophospholipids

Glycerophospholipids, alternatively known as phospholipids, are similar to neutral glycerolipids, consisting of up to two FAs attached to a glycerol backbone with a polar head group (Ref. 96). The polar head group of glycerophospholipids defines each subclass and can consist of a choline (phosphatidylcholine/PC), serine (phosphatidylserine/PS), glycerol (phosphatidylglycerol/PG), inositol (phosphatidylinositol/PI) or ethanolamine (phosphatidylethanolamine/PE) (Ref. 97). Glycerophospholipids are major membrane lipids, and their roles in signal transduction, organelle functions and pathophysiological processes are well-documented in the literature (Ref. 98). Significantly altered blood concentrations of glycerophospholipids in TBI, burn injury and SARS-CoV-2 infection are summarised in Table 3.

**Table 3.** Significantly altered glycerophospholipid concentrations compared to controls across blunt force trauma, thermal injury, and viral infection models

Subclass	Species	Force Trauma (TBI)	Thermal Trauma (Burn injury)	Viral Infection (SARS-CoV-2)
Phosphatidylcholine (PC)	PC 18:2_18:2	NR	↓ (Ref. 14)	↓ (Ref. 51)
	PC 18:0_18:2	NR	↓ (Ref. 14)	↓ (Ref. 51)
Phosphatidylglycerol (PG)	NR	NR	NR	NR
Phosphatidylserine (PS)	NR	NR	NR	NR
Phosphatidylinositol (PI)	PI 18:0_18:0	NR	↓ (Ref. 14)	↓ (Ref. 51)
	PI 18:0_20:4	NR	↓ (Ref. 14)	↓ (Ref. 51)
	PI 18:1_18:2	↓ (Ref. 66)	NR	↓ (Ref. 51)
	PI 20:0_18:2	↓ (Ref. 66)	↓ (Ref. 14)	↓ (Ref. 51)
Phosphatidylethanolamine (PE)	PE 16:0_18:3	NR	↓ (Ref. 14)	↓ (Ref. 51)
	PE 18:0_18:1	NR	↑ (Ref. 99)	↓ (Ref. 50)
	PE 18:1_18:1	NR	↑ (Ref. 99)	↓ (Ref. 51)
	PE 18:1_18:2	NR	↓ (Ref. 14)	↓ (Ref. 51)
	PE 18:1_20:4	↑ (Ref. 66)	NR	↓ (Ref. 50)
Lysophosphatidylcholine (LPC**)**	LPC 18:2	↑ (Ref. 27)	NR	↓ (Ref. 51)
	LPC 20:2	↓ (Ref. 27)	NR	↓ (Ref. 51)
	LPC 20:4	↓ (Refs 25, 26)	NR	↑ (Ref. 50)
Lysophosphatidylethanolamine (LPE)	LPE 18:1	NR	↑ (Ref. 99)	↓ (Ref. 51)

*Note:* ↑: increased levels; ↓: decreased levels; NR: not reported in cited literature; PC: phosphatidylcholine; PG: Phosphatidylglycerol; PS: Phosphatidylserine; PI: phosphatidylinositol; PE: phosphatidylethanolamine; LPC: Lysophosphatidylcholine; LPE: Lysophosphatidylethanolamine. To streamline the focus of this review, lipidome alterations observed in two or more insult types are tabularised above.

#### Phosphatidylcholin

The PC subclass is one of the most abundant phospholipids in the human body and a significant component of biological membranes and very low-density lipoproteins (Ref. 100). PC 18:0_18:2 and PC 18:2_18:2 decreased in burn injury (Ref. 14) and SARS-CoV-2 infection (Ref. 51), yet no evidence of significant alterations following TBI was identified in the literature. Research highlights the common trend of PC species decreasing in concentration following burn injury (Ref. 14), or SARS-CoV-2 infection. Further research into the behaviour of PC species following TBI may further support this common decrease in concentration. Treede et al. characterised chronic inflammatory disorder of the colon with low levels of PCs. In a follow-up in vitro study on cells and phagosomes, increased levels of PC inhibit proinflammatory mechanisms (Ref. 101). These findings suggest that the trending decrease in PC species across two acute insult exemplars facilitates the proinflammatory environment characterising burn injury and SARS-CoV-2 infection.

#### Phosphatidylglycerol

PG lipid species are sequentially synthesised from cytidine diphosphate DG in minimal amounts, constituting only 1–2% of all phospholipid species within cell membranes. The literature reports PG species to be downregulated in burn injury (PG 16:0_18:1) (Ref. 14) and SARS-CoV-2 patients (PG 20:0_18:1, PG 20:0_18:2 and PG 20:0_20:3) (Ref. 51). No PG species have been reported in TBI. In 2018, Chen et al. reported that supplementation with PG 18:1_18:1 and 18:2_18:2 in RAW264.7 cells attenuated inflammation by reducing prostaglandin-endoperoxide synthase 2 (PTGS2) messenger RNA expression, a key component of the PTGS2/COX2-PGE2 signalling cascade (Ref. 102). The downregulation of PG species in both burn injury and SARS-CoV-2 infection suggests a potential role of these lipids in the systemic response to acute insult. Whether that be the absorption of PG species from circulating blood to cellular structures or as part of a broader regulatory mechanism in inflammation.

#### Phosphatidylserine

PS species account for ~2–15% of phospholipids in mammalian physiology and are biosynthesised via the base-exchange reactions of PC and PE (Ref. 103). PS 18:0_18:2 was downregulated in burn injury (Ref. 14), while in TBI, PS 14:0_18:2 (Ref. 66) and PS 16:0_20:4 (Ref. 27) were reported to increase significantly in patients. No significant alterations in PS metabolism were reported in SARS-CoV-2 patients. In 2022, Darabi et al. characterised PS as a potent anti-inflammatory component in PC/PS-reconstituted high-density lipoprotein (rHDL)-treated mice, where IL-6 levels were significantly reduced compared to PC-rHDL-treated mice (Ref. 104). Due to the highly inflammatory nature of burn injury and TBI, the increase in PS species following TBI may be due to damage to PS-rich brain structures, releasing PS species across the blood–brain barrier.

#### Phosphatidylinositol

PI species are crucial for intracellular signalling and protein/carbohydrate anchoring to outer cell membranes. The biosynthesis of PI occurs via the transfer of an alcohol group from cytidine diphosphate diacylglycerol (CDP-DG) to inositol, facilitated by CDP-alcohol phosphotransferases (Ref. 105). PI 18:0_18:0 and 18:0_20:4 concentrations are reported to decrease in both burn injury (Ref. 14) and SARS-CoV-2 infection (Ref. 51). However, there is no evidence to support significant alterations of this lipid species in TBI. PI 18:1_18:2 concentrations decreased across TBI (Ref. 66), burn injury (Ref. 14) and SARS-CoV-2 infection (Ref. 51). The reported significant findings demonstrate that the PI species containing a linoleic acid (FFA 18:2) sidechain decreased across all three trauma types, indicating a disruption in signalling mechanisms. Furthermore, PI derivative PI 4,5-bisphosphate (PIP2) is a key regulatory component in the inflammatory response. The PIP2 molecule acts as a precursor for phospholipase C (PLC), which results in the hydrolysis of DG, an activator of protein kinase C and inositol(1,4,5)trisphosphate (IP3/I(1,4,5)P3), which facilitates an increase in intracellular calcium (Ref. 106). Systemic inflammation is hallmarked by a calcium surplus, resulting in endothelial cell injury (Ref. 107).

#### Phosphatidylethanolamine

PE biosynthesis occurs via four pathways: the CDP-ethanolamine pathway, the acylation of lyso-PE, exchange reactions and the PS decarboxylase pathway (Ref. 108). The PE lipid class is a precursor for PC and influences autophagy, cell and organelle membrane fusion and oxidative phosphorylation (Ref. 108). The concentrations of PE species PE 16:0_18:3 and PE 18:1_18:2 are reported to decrease in burn injury (Ref. 14) and SARS-CoV-2 infection (Ref. 51). PE 18:1_20:4 concentrations significantly increased following TBI (Ref. 66), while significantly decreased in SARS-CoV-2 (Ref. 50). The discrepancy between species decreasing in burn injury and SARS-CoV-2 infection, yet increasing in TBI, highlights the complex, context-dependent roles of the PE class in different conditions. These findings suggest that PE species may have distinct functions, depending on the nature of the injury or disease.

#### Lysophosphatidylcholine

Lysophosphatidylcholine (LPC) is derived from the cleavage of PC via the action of phospholipase A2 or by the transfer of FAs to free cholesterol through lecithin-cholesterol acyltransferase (Refs 109, 110). LPC has various effects on different cell types; please refer to (Ref. 111) for a thorough review. The literature reports divergent responses post-traumatic insult, with reports of increases in LPC 18:2 in response to TBI but a decrease following SARS-CoV-2 infection. This observation may indicate a specific response dependent on the type of inflammatory response, a finding supported by reports that LPC 18:2 has a negative association with the systemic immune inflammation index, the neutrophil-lymphocyte ratio and the platelet-lymphocyte ratio (Ref. 112), indicating links to immune activation.

A second divergent observation is the reports of LPC 20:4, which is decreased in TBI patients (Refs 25, 26) but increased in SARS-CoV-2 infected patients (Ref. 50). Mechanistic studies indicate that LPC 20:4 has anti-inflammatory properties in response to LPC 16:0-induced inflammation, which results in the downregulation of leukocyte-associated damage and up-regulation of anti-inflammatory mediators (IL-4 and IL-10) (Ref. 113). These findings suggest that increased levels of LPC 20:4 may result from modulation of inflammation.

#### Lysophosphatidylethanolamine

Lysophosphatidylethanolamine (LPE) accounts for ~1% of total phospholipids in the blood and is biosynthesised through the cleavage of a single FA side chain from PE via phospholipase A1/A2 (Ref. 114). While LPEs have been studied in relation to systemic response to trauma, there is minimal overlap of specific species reported in the literature (Table 3). However, LPE trends appear inconsistent across studies, which complicates their interpretation. LPE 20:1 (Ref. 66) and 20:4 (Ref. 25) concentrations are reported to decrease in TBI, while LPE 18:0 and LPE 18:1 increase in burn injury (Ref. 99). The LPE response in SARS-CoV-2 infection exhibits many discrepancies. LPEs 16:0 and 18:2 are reported to increase (Refs 50, 51) and decrease (Refs 52, 115) in response to infection. These conflicts underscore the challenges of comparing literature due to the varied study designs employed.

### Sphingolipids

Sphingolipids account for ~10% of total lipids in mammals (Ref. 116). While all other lipid classes consist of a glycerol backbone, sphingolipids uniquely have a sphingosine backbone (Ref. 117). Key sphingolipids include ceramides (CERs) and sphingomyelins (SMs). CERs are precursors for the synthesis of sphingosine 1-phosphate and CER 1-phosphate, which have a vital role in signalling for apoptosis, autophagy and activation of the NLR family pyrin domain containing 3 inflammasome to increase production and secretion of proinflammatory cytokines IL-1β and IL-18 (Ref. 118). Reports of significantly altered blood concentrations of sphingolipid species in TBI, thermal burn injury and SARS-CoV-2 viral infection are summarised below (Table 4).

**Table 4.** Significantly altered sphingolipid concentrations compared to controls across blunt force trauma, thermal injury, and viral infection models

Subclass	Species	Force Trauma (TBI)	Thermal Trauma (Burn injury)	Viral Infection (SARS-CoV-2)
Ceramide (CER)	CER d18:1_22:0	↓ (Ref. 27)	NR	↑ (Refs 119, 120)
Dihydroceramide (DCER)	NR	NR	NR	NR
Hexosylceramide (HCER)	HCER 22:0	↑ (Ref. 66)	NR	↓ (Ref. 54)
Sphingomyelin (SM)	NR	NR	NR	NR

*Note:* ↑: increased levels; ↓: decreased levels; NR: not reported in cited literature; CER: Ceramide; DCER: Dihydroceramide; HCER: Hexosylceramide; SM: Sphingomyelin. To streamline the focus of this review, lipidome alterations observed in two or more insult types are tabularised above.

The literature contains many examples of significant sphingolipid alterations in the blood following SARS-CoV-2 infection (COVID-19) (Refs 51, 115, 119, 120). In contrast, there is far less evidence describing comparable changes after TBI (Refs 27, 30) or burn injury (Refs 14, 36, 40, 99). We speculate that the surge of funding, availability of large sample populations and intense research focus on COVID-19 during the early 2020s may have led to a gap in the depth of lipidomic research across the exemplars, as demonstrated by the significantly lower number of publications in burn injury and TBI lipidomics. While this disparity may partly reflect the lower incidence of TBI and burn injury compared with COVID-19, this review demonstrates clear value in studying these conditions. We encourage researchers to expand lipidomic investigations in both the burn injury and TBI fields.

#### Ceramide

CER is biosynthesised by the reduction of dihydroceramide via dihydroceramide desaturase in the ER (Ref. 121). The CER lipid class comprises bioactive molecules that are pivotal in vital signalling pathways responsible for controlling cell differentiation and proliferation (Ref. 122). Evidence in the literature demonstrates that concentrations of CER d18:1_22:0 decrease in TBI (Ref. 27), yet an increase in SARS-CoV-2 infection. The literature does not characterise the specific role of CER d18:1_22:0 in inflammation. However, in 2022, McNally et al. demonstrated that palmitic acid-induced cell stress causes an increase in the secretion of CER d18:1_22:0 through the activation of protein kinase R-like ER kinase pathway (Ref. 123), a key regulator of protein synthesis during ER stress, which is often seen in inflammatory disease (Ref. 124). Interestingly, palmitic acid was reported to increase in all models of trauma (see Palmitic acid subsection for further information). These findings suggest that ER stress may be lower in blunt force trauma compared to viral infection.

#### Hexosylceramide

Hexosylceramide (HCER) is produced via the action of HCER synthase on CER and is a precursor for gangliosides, a significant class of signalling molecules throughout the nervous system (Ref. 125). Anyaegbu et al. TBI study reported an increase in HCER 22:0 (Ref. 66). In contrast, Lodge and colleagues demonstrated that SARS-CoV-2 patients have reduced concentrations of HCER 22:0 across all severity levels when compared to controls (Ref. 54). The lack of a defined sphingoid base in the naming convention makes it implausible to conclude that these are the same features. While the exact role of HCER species in SARS-CoV-2 infection and TBI remains to be elucidated, HCER d18:1_22:0 significantly decreases in early-stage alcoholic liver disease, a condition known to induce proinflammatory conditions in the liver and proposed as a therapeutic target (Ref. 126). Furthermore, Zhu and colleagues demonstrated that HCER d18:1_22:0 is strongly positively associated with the systemic immune-inflammation index in a study examining the relationship between plasma metabolites and inflammatory biomarkers in coronary artery disease (Ref. 112). These findings suggest that HCER has a strong relationship with inflammation and potentially highlight HCER species as proinflammatory in systemic inflammation following an acute insult.

#### Sphingomyelin

SM is biosynthesised from CER by the enzymatic reaction of SM synthase and functions as a secondary lipid messenger and a stage for signalling molecules (Refs 121, 127). The evidence for significantly altered SM concentrations at the time of this review was minimal for TBI and burn injury. However, available evidence indicates that SM concentrations decreased in SARS-CoV-2 infection (Ref. 120) and TBI (Ref. 27). In an inflammatory environment, the upregulated production of sphingomyelinases converts SM into CER and phosphocholine (Ref. 128). These findings indicate that SM conversion to CER and phosphocholine is increased in TBI and SARS-CoV-2 infection; however, further research with a broader range of SM species is needed to confirm this observation.

### Lipoproteins

Lipoproteins facilitate the transport of lipids in plasma, functioning as essential carriers. This dynamic interplay is essential for cellular homeostasis and overall physiological well-being (Ref. 129). Lipoproteins are categorised based on size (nm), density (g/ml) and lipid composition into the following classes: very low density (0.950–1.006 kg/L), intermediate density (1.006–1.019 kg/L), low density (1.019–1.063 kg/L), high density (1.063–1.210 kg/L) and chylomicrons (Ref. 130). Research in the lipoprotein niche for TBI and burn injury is limited, with very few research papers reporting specific lipoprotein species concentrations instead of overall class concentrations. Conversely, the literature is saturated with studies on the impact of SARS-CoV-2 on lipoproteins, driven by the global pandemic and the worldwide effort to understand the severe nature of SARS-CoV-2 infection. These observations are presented in Table 5 below.

**Table 5.** Significantly altered lipoprotein concentrations compared to controls across blunt force trauma, thermal injury, and viral infection

Subclass	Species	Force Trauma (TBI)	Thermal Trauma (Burn injury)	Viral Infection (SARS-CoV-2)
Very Low-Density Lipoproteins (VLDL)	V5PL	NR	↑ (Ref. 14) ↓ (Ref. 131)	↑ (Refs 54, 132)
	V5CH	NR	↑ (Ref. 14) ↓ (131)	↑ (Refs 54, 132)
	V5FC	NR	↓ (Ref. 131)	↑ (Refs 54, 132)
Low-density lipoproteins (LDL)	L1TG	NR	↓ (Ref. 131)	↓ (Refs 54, 132)
	L2TG	NR	↑ (Ref. 14)	↓ (Refs 54, 132)
	L4TG	NR	↓ (Ref. 131)	↓ (Refs 54, 133)
	L6TG	NR	↑ (Ref. 131)	↓ (Refs 54, 132)
	L2CH	NR	↑ (Ref. 14)	↑ (Refs 54, 132)
	L5CH	NR	↑ (Ref. 131)	↓ (Refs 54, 132)
	L2PL	NR	↑ (Ref. 14)	↓ (Refs 54, 132)
	L2FC	NR	↑ (Ref. 14)	↓ (Refs 54, 132)
High-density lipoproteins (HDL)	HDA1	NR	↓ (Ref. 14)	↓ (Refs 54, 133)
	H2A2	NR	↑ (Ref. 131)	↓ (Refs 54, 132)
	H4A1	NR	↓ (Ref. 14)	↓ (Refs 54, 133)
	H4FC	NR	↓ (Ref. 14)	↓ (Refs 54, 133)
	H4CH	NR	↓ (Ref. 14)	↓ (Refs 54, 133)

*Note:* ↑: increased levels; ↓: decreased levels; NR: not reported in cited literature; V5PL: Very Low-Density Lipoprotein-5 Subclass Phospholipids; V5CH: Very Low-Density Lipoprotein-5 Subclass Cholesterols; V5FC: Very Low-Density Lipoprotein-5 Subclass Free Cholesterols; L1TG: Low-Density Lipoprotein-1 Subclass Triglycerides; L2TG: Low-Density Lipoprotein-2 Subclass Triglycerides; L3TG: Low-Density Lipoprotein-3 Sub-class Triglycerides; L4TG: Low-Density Lipoprotein-4 Subclass Triglycerides; L6TG: Low-Density Lipoprotein-6 Subclass Triglycerides; L2CH: Low-Density Lipoprotein-2 Sub-class Cholesterols; L5CH: Low-Density Lipoprotein-5 Subclass Cholesterols; L2PL: Low-Density Lipoprotein-2 Subclass Phospholipids; L2FC: Low. Density Lipoprotein-2 Sub-class Free Cholesterol; HDA1: HDL Apolipoprotein A-I; H2A2: High-Density Lipoprotein-2 Subclass Apolipoprotein-A2; H4TG: High-Density Lipoprotein-4 Subclass Triglycerides. To streamline the focus of this review, lipidome alterations observed in two or more insult types are tabularised above.

#### Very-low-density lipoproteins

Very-low-density lipoproteins (VLDL) are triglyceride-rich lipoproteins consisting of cholesterol esters, phospholipid membranes and apolipoproteins B-100, C-I, C-II, C-III and E (Ref. 134). VLDL particles are synthesised in the liver and released into the bloodstream, where they undergo lipolysis by lipoprotein lipase, resulting in VLDL remnants (Ref. 134). VLDL plays a crucial role in transporting triglycerides and cholesterol from the liver to peripheral tissues. In 2023, Ryan et al. demonstrated that burn injury patients display increased VLDL subfractions V5PL and V5CH (Ref. 14). The upregulation of V5PL and V5CH was mirrored in studies by Lodge et al. and Masuda et al. on SARS-CoV-2 infection (Refs 54, 132). Furthermore, 22 VLDL subfractions were significantly altered in patients with SARS-CoV-2 infection: 17 VLDL increased in concentration, and 5 decreased. Notably, VLDL subfractions free cholesterol (VLFC, V3FC) and cholesterol (V2CH, V3CH, V4CH) were significantly decreased (Refs 54, 132). In contrast, Begum and colleagues reported decreased concentrations of V5PL, V5CH and V5FC in children 3 years post-burn injury compared to non-injured age and sex-matched controls, proposing a ‘metabolic memory’ of burn injury (Ref. 131). The discrepancies in V5PL and V5CH highlight the importance of standardising variables such as time from injury and age when undertaking cross-study comparisons.

Elevated VLDL, frequently observed in burn injury and SARS-CoV-2 infection, has been linked to increased conversion to IDL and LDL, resulting in higher atherosclerotic risk (Ref. 135). Increased VLDL concentrations have also been implicated in potentiating platelet activation and aggregation, contributing to the prothrombotic phenotype commonly observed in severe inflammatory states (Ref. 136). Barcena and colleagues (2022) utilised human monocytic cell line THP-1 cells in either a proinflammatory or anti-inflammatory state to deduce that treatment with human VLDL significantly suppressed the expression of inflammatory biomarkers TNF-α, cluster of differentiation 80 and IL-1β in proinflammatory cells (Ref. 137). In the context of these findings, the upregulation of VLDL in burn injury and SARS-CoV-2 may indicate a broader regulatory metabolic response to inflammation and tissue damage following an acute insult.

#### Low-density lipoproteins

Low-density lipoproteins (LDLs) are biosynthesised from VLDL in the circulatory system and carry the most cholesterol out of the lipoprotein classes. In addition, most LDL subfractions contain a single apolipoprotein B-100 (Ref. 134). Within the LDL class, size indicates a pathological tendency; increased concentrations of small, dense LDL are associated with inflammatory states and disease (Ref. 138). Interestingly, inverse perturbations were observed for LDL-2 subfractions (L2FC, L2PL and L2TG) and L5CH. Specifically, in burn injury, Ryan et al. demonstrated an increase in L2FC, L2PL, L2TG and L5CH (Ref. 14). In contrast, Lodge et al. and Masuda et al. demonstrated that SARS-CoV-2 infection decreased L2FC, L2PL, L2TG and L5CH (Refs 54, 132). Additionally, these authors reported that L2CH concentrations increased, while L1TG and L4TG concentrations decreased, in burn injury (Ref. 14) and SARS-CoV-2 infection (Refs 54, 132).

LDL remodelling similarly contributes to inflammatory dysfunction. Small, dense LDL particles, which increase in acute inflammatory conditions, exhibit greater susceptibility to oxidative modification (Ref. 139). Oxidised LDL (oxLDL) is a potent proinflammatory mediator that activates endothelial TLR and scavenger receptor signalling, enhances endothelial permeability and promotes monocyte adhesion and extravasation (Ref. 139). OxLDL also drives macrophage differentiation towards a proinflammatory state, increasing cytokine production, inflammasome activation and tissue-level inflammatory injury (Ref. 140). These mechanistic pathways underscore that LDL alterations observed in burn injury and SARS-CoV-2 infection may impact vascular inflammation beyond changes in lipid transport alone.

#### High-density lipoproteins

HDL particles, which are saturated in cholesterol and phospholipids, play a vital role in the process known as reverse cholesterol transport, facilitating the transport of cholesterol from peripheral tissues to the liver. Regarding apolipoprotein content, HDLs contain A-I, A-II, A-IV, C-I, C-II, C-III and E, with A-1 forming the primary protein structure for HDL particles (Ref. 134). In burn injury, HDL particles undergo significant remodelling, with studies by Ryan et al. reporting an increase in H2A2 and a concurrent decrease in HDL-4 subfraction cholesterol, free cholesterol, apolipoprotein A1 and HDA1, suggesting a stress-induced shift in HDL particle structure and function. Conversely, in SARS-CoV-2 infection, Lodge et al. and Masuda et al. observed a decrease in H2A2, HDA1, H4A1, H4FC and H4CH, indicating a suppression of HDL-associated proteins and lipids. These opposing trends in H2A2 expression, elevated in burns and reduced in COVID-19, highlight the context-dependent nature of HDL remodelling. Additionally, TBI has been associated with a class-wide reduction in total HDL levels, as reported in 2021 by Zhong et al. (Ref. 32), further underscoring HDL’s vulnerability to acute insults.

HDL is anti-inflammatory under physiological conditions, and reductions in HDL concentration or changes in HDL composition impair protective functions. During acute inflammation, HDL can become functionally compromised, adopting a dysfunctional state characterised by reduced cholesterol efflux capacity, diminished antioxidant activity and impaired ability to neutralise lipopolysaccharides and other inflammatory lipids (Ref. 141). Loss of HDL-associated enzymes such as paraoxonase-1 and alterations in apolipoprotein A-I can decrease HDL’s ability to inhibit LDL oxidation, further contributing to endothelial dysfunction and immune activation (Ref. 142). These changes likely explain the consistent finding of reduced HDL subclasses across burn injury, TBI and SARS-CoV-2 infection. Collectively, these findings emphasise a general trend in the reduction of HDL species following acute insults.

## Conclusions

The growing emphasis on personalised medicine and healthy ageing offers tremendous potential for improving public health outcomes. Nevertheless, a key challenge persists: despite widespread recognition of the inflammatory response in acute trauma, there remains a lack of definitive evidence supporting its systematic monitoring across different types of insults. This review highlights lipids and their associated lipoproteins as uniquely robust biomarkers for decoding the systemic response to trauma. These metabolic signatures provide detailed insights into how the body reacts to a range of insults from blunt force injuries like TBI, to thermal injury such as burns, and viral infections such as SARS-CoV-2. While these markers illuminate individual trauma responses, our understanding of how different insults converge or diverge in their inflammatory metabolic profiles is still limited.

To make the patterns clear across TBI, burn injury and SARS-CoV-2 infection and to link them to inflammatory mechanisms and biological insights, we described the lipid changes in a sequential order from FAs to glycerolipids, glycerophospholipids, sphingolipids and lipoproteins. This structure aimed to provide readers with a consistent scaffold for interpreting ‘increase/decrease’ directions despite heterogeneous reporting across studies. TBI shows hallmarks of membrane disruption and blood–brain barrier breach, including loss of structural phospholipids (notably PC and PI), release of PUFA substrates (including linoleic and arachidonic acids), divergent LPC signals consistent with immune activation and repair and a general reduction in HDL, all of which align with neuroinflammation. Burn injury literature was dominated by tissue loss and oxidative stress, which together explain broad depletion of PC/PI/PE, sustained elevations in palmitic and stearic acids that drive lipotoxic and inflammatory signalling, increases in MG/TG species and an acute rise in VLDL (and some LDL2) consistent with hepatic export and lipid transport demands. SARS-CoV-2 infection presents a cytokine-driven hepatic and adipose rewiring and viral use of lipid microdomains, with frequent decreases in PC/PI/PE and MG/DG, pronounced sphingolipid remodelling (increased CERs, decreased HCERs and SMs) and increased VLDL and decreased HDL. Situating these insults within a shared biological framework underscores the extent to which their lipidomic signatures overlap.

Considered together, the lipidomic profiles of TBI, burn injury and SARS-CoV-2 converge on a common inflammatory metabolic signature. For example, rising saturated FAs (palmitic acid and stearic acid), depletion of key phospholipids (especially linoleoyl-containing PC/PI), HDL depression and VLDL elevation. Conversely, the same lipidomic profiles diverge in specific ways, including MG/DG directionality (higher in TBI/burn injury but lower in SARS-CoV-2), arachidonic acid (lower in burn injury but higher in TBI/SARS-CoV-2), LPC 20:4 (lower in TBI but higher in SARS-CoV-2) and a stronger sphingolipid concentration in SARS-CoV-2. Framed this way, the observed increases and decreases across classes start to map onto coherent biological pathways. For example, TLR mediated lipotoxic signalling and ER stress, membrane turnover and repair, eicosanoid biosynthesis from PUFAs and lipoprotein mediated redistribution. Providing a rationale for translational, lipidomic panels (e.g., palmitate, PC/PI indices, LPC 20:4, selected CERs and VLDL/HDL subfractions) to stratify patients across insult types.

Metabolic phenotyping, including lipidomics and lipoproteins, can meaningfully inform care after acute injury and infection, but progress hinges on reducing methodological variability. We advocate for harmonised, multi-insult, longitudinal studies that standardise pre-analytics and platforms to distinguish convergent from divergent lipid and lipoprotein perturbations. Priorities should include integrative preclinical models that pair lipidomics with other omics to test mechanisms, and clinical trials that target lipid pathways while embedding lipidomic endpoints. To ensure transportability, studies should harmonise metadata capturing: age, biological sex and hormonal status (e.g., menopausal state and hormone therapy), relevant medications and tightly controlled pre-analytics (collection-to-spin time, storage conditions, freeze–thaw count and use shared QC materials across sites). In parallel, transferable biomarker panels should be advanced through analytical and clinical validation for prognosis at presentation and for monitoring treatment response. Together, these steps can translate robust lipid signatures into pragmatic tools that improve triage, personalise interventions and ultimately enhance patient outcomes.

## List of abbreviations

AP-1: Activator protein-1
ApoA-I: Apolipoprotein A-I
ApoA-II: Apolipoprotein A-II
ApoB-100: Apolipoprotein B-100
ApoC-I: Apolipoprotein C-I
ApoC-II: Apolipoprotein C-II
ApoC-III: Apolipoprotein C-III
ApoE: Apolipoprotein E
CCL2: C–C motif chemokine ligand 2 (MCP-1)
CCL3: C–C motif chemokine ligand 3 (MIP-1α)
CER: Ceramide
COX-2: Cyclooxygenase-2
COVID-19: Coronavirus disease 2019
CRP: C-Reactive protein
DCER: Dihydroceramide
DG: Diacylglycerol
ER: Endoplasmic reticulum
ESR: Erythrocyte sedimentation rate
FA: Fatty acid
FFA: Free fatty acid
HCER: Hexosylceramide
HDL: High-density lipoprotein
HDA1: HDL apolipoprotein A-I (aggregate measure)
H2A2: HDL-2 apolipoprotein A-II
H4A1: HDL-4 apolipoprotein A-I
H4CH: HDL-4 cholesterol
H4FC: HDL-4 free cholesterol
H4TG: HDL-4 triglycerides
I(1,4,5)P3: Inositol(1,4,5)trisphosphate (see IP3)
IL: Interleukin
IL-1: Interleukin-1
IL-1β: Interleukin-1 beta
IL-4: Interleukin-4
IL-6: Interleukin-6
IL-10: Interleukin-10
IP3: Inositol 1,4,5-trisphosphate
IRF3: Interferon regulatory factor 3
LDL: Low-Density lipoprotein
L1TG: LDL-1 triglycerides
L2CH: LDL-2 cholesterol
L2FC: LDL-2 free cholesterol
L2PL: LDL-2 phospholipids
L2TG: LDL-2 triglycerides
L3TG: LDL-3 triglycerides
L4TG: LDL-4 triglycerides
L5CH: LDL-5 cholesterol
L6TG: LDL-6 triglycerides
LPC: Lysophosphatidylcholine
LPE: Lysophosphatidylethanolamine
MAPK: Mitogen-activated protein kinase
MG: Monoacylglycerol
MIP-1α: Macrophage inflammatory protein-1 alpha (CCL3)
MyD88: Myeloid differentiation primary response 88
NF-κB: Nuclear factor kappa-light-chain-enhancer of activated B cells
NLRP3: NOD-like receptor family pyrin domain containing 3
NR: Not reported (in cited literature)
oxLDL: Oxidised low-density lipoprotein
PC: Phosphatidylcholine
PE: Phosphatidylethanolamine
PG: Phosphatidylglycerol
PGE1: Prostaglandin E1
PGE2: Prostaglandin E2
PI: Phosphatidylinositol
PIP2: Phosphatidylinositol 4,5-bisphosphate
PKC: Protein kinase C
PL: Phospholipids (suffix in lipoprotein subfractions)
PON1: Paraoxonase-1
PPARα: Peroxisome proliferator-activated receptor-alpha
PPARβ/δ: Peroxisome proliferator-activated receptor-beta/delta
PSD: (Psd) Phosphatidylserine decarboxylase
PTGS2: Prostaglandin-endoperoxide synthase 2
PUFA: Polyunsaturated fatty acid
RAW264.7: Mouse macrophage cell line
rHDL: Reconstituted high-density lipoprotein
ROS: Reactive oxygen species
SARS-CoV-2: Severe acute respiratory syndrome coronavirus 2
SIRS: Systemic inflammatory response syndrome
SM: Sphingomyelin
TBI: Traumatic brain injury
TG: Triacylglycerol (triglyceride)
THP-1: Human monocytic cell line
TLR2: Toll-like receptor 2
TLR4: Toll-like receptor 4
TNF: Tumour necrosis factor
TNF-α: Tumour necrosis factor alpha
VLDL: Very-low-density lipoprotein
V2CH: VLDL-2 cholesterol
V3CH: VLDL-3 cholesterol
V4CH: VLDL-4 cholesterol
V3FC: VLDL-3 free cholesterol
V5CH: VLDL-5 cholesterol
V5FC: VLDL-5 free cholesterol
V5PL: VLDL-5 phospholipids
VLFC: VLDL free cholesterol
